# The Evacuation of Wounded from Forward Areas

**Published:** 1918-09

**Authors:** H. Ensor


**Methods Employed During the War for the Evacuation of Wounded from Forward Areas.** Colonel H. Ensor.

The methods of transportation of wounded, the speaker said, have undergone considerable improvement since the outbreak of war.

At that period the methods were the following : — The horsed ambulance wagons attached to each field ambulance conveyed the wounded from as near to the firing line as possible to the divisional dressing stations. From the latter point they were removed to the casualty clearing stations or clearing hospitals by means of the G. S. wagons of the supply sections of the divisional train and the motor lorries of the divisional supply column.

Supplies reached a division in the field in the following manner : —

A place on a railway line was chosen as the railhead of the division, where a casualty clearing station, or part of one, was opened. The supplies arrived by train and were loaded into the motor lorries of the divisional supply column and taken to a place known as the refilling point. At this point the G. S. wagons of the supply sections of the divisional train met the motor lorries of the supplies column and the rations, etc., were transferred to the G. S. wagons which delivered them to the troops in the field.

This routine was carried out every twenty-four hours, the important fact being that once during these twenty-four hours the G. S. wagons returning empty from the divisional area to the refilling point, and the motor lorries of the supply column returning empty from the divisional train to the railhead, were available for the transportation of sick and wounded from the divisional area to the refilling point, and from there to the railhead casualty clearing station where they received attention until evacuation to the base hospitals by means of ambulance trains was possible.

The railhead and the refilling point varied in accordance with the movements of the division, and with the railhead, the casualty clearing station, and the supply column headquarters. The refilling point must never be more than 45 miles from the railhead and, as a rule, it was very seldom more than 15.

The speaker next considered how the A. D. M. S. of the division succeeded in getting the wounded away from the railhead.

He took care that the main dressing station was situated on a road which the G. S. wagons of the supply section of the train would use on their way to the refilling point.

The A. A. and the Q. M. G. gave orders to the O. C. train that his G. S. wagons were to halt at the dressing station for the purpose of taking up the sick and wounded and carrying them to the refilling point.

The O. C. supply column would also receive similar orders to transport the patients to the casualty clearing station at the railhead.

These early arrangements seem crude and imperfect to us now, but they worked well so far as the actual evacuation of the sick and wounded was concerned.

The advantage of this system of evacuation was its economy of transport. Its disadvantages were many and obvious. The most important were: (1) The unsuitability of the G. S. wagons and 3-ton lorries, no matter how carefully prepared with straw and hay, for the carriage of severely wounded and dangerously ill men. (2) The dressing stations could only be cleared once in twenty-four hours instead of continuously as at present. (3) The medical services were dependent upon transport of which they had not sole control, and of which the primary duty was the feeding of troops. (4) The evacuation of wounded from the dressing stations to the refilling point was very slow and often took hours.

Steps were taken to render the medical services independent of the supply services by providing motor ambulance cars. The division the speaker was with was provided with two such cars. In a time of stress they succeeded in clearing quite two-thirds of the lying-down cases.

By the end of October 1914, or early in November, every army corps had been provided with a motor ambulance convoy, and these cleared individual dressing stations into the casualty clearing stations daily or as often as was necessary. This rendered it unnecessary for the clearing stations to be attached to the railheads, and they were opened out at other convenient places where good accommodation was available.

Early in 1915, seven motor Ambulance cars were allotted to each field ambulance of a division, and the seven horsed ambulance wagons were withdrawn. This is the present establishment of each divisional field ambulance in the B. E. F.

In the pursuit of a defeated enemy, horse rather than motor transport will prove advantageous in that it can more easily surmount the obstructions which the enemy is sure intentionally to leave in his wake.

At the present time, the evacuation of the wounded is carried out as follows: —

The A. D. M. S. of an attacking division reserves all available motor transport (15 large ambulance cars and 6 Fords) for the lying-down and sitting cases. The Fords are on duty in front of the advanced dressing station, so as to relieve the field ambulance bearers, whereas the large cars will do the work from the advanced dressing station to the main dressing station.

The large cars will then act as a divisional convoy. The time of assembly of the cars will have to be some hour after dark on the day before the attack, or else before daybreak on the day of the attack, in order to avoid being seen by the enemy's aircraft.

When all is ready for the attack, there ought to be one car on duty at each advanced dressing station. At a given point on each road leading from each advanced dressing station there should be a cab-rank of three cars. The remaining cars will be in readiness at the headquarters of the divisional motor ambulance convoy. As soon as the first is seen to leave the advanced dressing station, one of the cab-rank moves forward to replace it, and is in turn replaced by one of the cars from headquarters. The driver as he comes in must not fail to state from which station he has come. This forms a constant stream of cars, and also gives the drivers a certain amount of time; while waiting to replace the car in front, for rest and food.

The duty of clearing the main dressing stations devolves upon the A. D. M. S. of the Army Corps to which the division belongs.

The result of the economy of time permitted by this new scheme is that within two or three hours after being wounded, the patients arrive at the casualty clearing station in a good condition to stand operation.

The slight walking cases, after being dressed at the regimental aid posts, are directed to a collecting station for walking wounded, from whence they are conveyed by motor lorry to a casualty clearing station.

Arrangements are usually made for any lorries which may happen to be proceeding in the required direction to be made available for slight cases. A small but important point in such arrangements is the provision of a ladder to assist the patients to mount.

